# Public health approaches to control injuries 

**Published:** 2019-08

**Authors:** Maryam Baradaran Binazir

**Affiliations:** ^*a*^Social Determinants of Health Research Center, Health Management and Safety Promotion Research Institute, Tabriz University of Medical Sciences, Tabriz, Iran.

## Abstract

**Background:**

Injuries are a focus of public health practice, because they pose a serious health threat. Injuries ‎are preventable in most situations. Despite successes in many areas of injury prevention, the ‎potential of preventing traumatic injuries has not been realized. There remains much to be done. ‎The aim of this study was to investigate different public health approaches to injury control by ‎article review.‎

**Methods:**

In the present review article, reputable internet databases were investigated. Studies that ‎considered models of public health in injuries control were included in this review. The ‎following databases were accessed for the period from beginning to 2018 for English ‎publications: Google Scholar, PubMed, Scopus and Science Direct .The terms “public health ‎models” and “injury” were used. Articles had to meet the following inclusion criteria: focus on ‎public health models and focus on injuries. The articles were critically appraised by using tools ‎developed by the Critical Appraisal Skills Program (CASP). The models were revealed by the ‎literature reviewed, summarized for data synthesis. ‎

**Results:**

From the literature review, we found two models in this area. First one is the causal model for ‎traumatic injuries. It is easily adopted from the traditional epidemiologic causal model. The ‎second one is the public health model. It advocate a cycle of surveillance, risk factor ‎identification, intervention implementation and evaluation. ‎

**Conclusions:**

The public health model and the causal model for traumatic injuries are sustainable framework ‎to use in injury control and prevention.‎

**Keywords:**

Injury, Public health, Model

